# Cross-cultural differences in retracted publications of male and female from a global perspective

**DOI:** 10.1007/s11192-023-04717-2

**Published:** 2023-05-14

**Authors:** Shenghui Li, Wenyan Xu, Jingqi Yin

**Affiliations:** 1 School of Public Administration, South China University of Technology, Guangzhou, 510640 China; 2 School of Sociology, Shanghai Normal University, Shanghai, 200233 China; 3 South China University of Technology, Wushan Road, Tianhe District, Guangzhou, 510641 Guangdong China

**Keywords:** Retracted publication, Publication integrity, Qualitative comparative analysis, Cross-cultural analysis, Gender difference, 91C99

## Abstract

The aim of this paper is threefold: (i) to identify the combinations of national culture dimensions that lead to high (or low) male or female retracted publications, (ii) to understand the role of personal trust as a relevant condition that combines with national culture dimensions to cause high (or low) male or female retraction, and (iii) to identify the differences in the configurations that lead to those outcomes. Based on framework of Hofstede's cross-cultural analysis and data from Hofstede Center, World Value, and Web of Science, this essay analyzes cultural complex causal relations between national culture and trust dimensions (conditions), and male and female retracted publications (outcomes) in 30 countries nationally and globally by fuzzy-set qualitative comparative analysis. This research provides three major insights: (i) Cultural dimensions (power distance, individualism, masculinity, uncertainty avoidance, long-term orientation) and trust are not necessary conditions for both male and female to cause retractions, (ii) different levels of personal trust (high/low) combine with national cultural dimensions in order to produce different configurations that can lead to high or low retractions, and (iii) Each gender causes retractions in a similar or identical way, but each also owns its unique way. Finally, we provide effective policy recommendations to specific countries based on our critical conclusions and discussions.

## Introduction

Over the past few decades, retracted publications (RP) have been increasingly recognized as a key factor affecting the scientific development of a country. A frequent occurrence of RP incidents in academic settings around the world has been a challenging issue. The number and frequency of retractions reflect the health of scientific undertakings, but this indicator is gradually increasing in recent years (Steen et al., 2013). Many authors from multiple countries have been found to be involved in unethical publishing practices (Amos, 2014), including India (Mukhopadhyay et al., 2023), Malaysia (Aspura et al., 2018) and other countries.

The first notice of retraction occurred in the 1970s. The frequency of retracted articles has risen dramatically since then, especially in the last 20 years (Cokol et al., 2008; Fang et al., 2012). According to available research, retracted publications show three obvious characteristics. Firstly, regarding the nature of the retractions, most of the retractions can be viewed as typical deliberate fraud (Wager & Williams, 2011). Plagiarism, fraud, and falsified peer review account for roughly three-quarters of retractions of papers, which explains the real-world scenarios in which retractions occur (Lei & Zhang, 2018). And retractions are mostly due to the ethical misconduct, authors misconduct or publishing misconduct (Aspura et al., 2018; Bar-Ilan & Halevi, 2018; Elango et al., 2019; Fang et al., 2012; Grieneisen & Zhang, 2012). Secondly, the scope of retractions and the subjects covered are highly varied. This phenomenon has spread across all disciplines, from the natural sciences to the social sciences. Whether it be in traditional developed countries like the United States, the United Kingdom, and Japan, as well as developing countries like India and China, there have been numerous journals and multiple researchers retracting articles (Vuong et al., 2020). Finally, retracted papers are causing concerns and worries among scholars in academia (Ataie-Ashtiani, 2017). The phenomenon exist in community is some researchers continue to cited highly retracted papers, which still available through online resources (Rzymski, 2021; Silva & Nszki, 2017).

Exploring cultural differences of retractions across countries contributes to understanding of publication ethics and integrity, investigating the difference between male and female, however, offered a different perspective. Several studies have explained that macro factors of national culture (Mousavi & Abdollahi, 2020), or trust between people in the social environment has an effect on RP (Madlock-Brown & Eichmann, 2015). Given the nature of responsible conduct in research, the research community needs to make attempts to control the impact of retracted papers between gender. Men and women in different countries to present high or low RP, owing to differences in population, system, social, cultural and other factors. The decision to focus on macro-level factors was made by scholars who believe that social, economic, cultural, and political environment shape individual behavior (Henrich, 2015). As a result, the environment influences scientists' behavior, expectations, and motivations. It is possible to evaluate the differences in scientific research environment between nations by describing the national culture and trust dimension. The national cultural dimension and trust of social environment are determinants of RP in males and females, according to this study.

This essay aims to conduct a comparative study on national culture and trust causing RP, using the fuzzy-set qualitative comparative analysis method (fsQCA) towards 30 countries in order to contribute to existing literature. The main objectives of this study are: (i) to determine the specific national cultural dimensions or the combination of national cultural dimensions that lead to high (or low) RP for men and women, (ii) to understand the role of trust as a relevant condition, combined with the national cultural dimensions, that contribute to high and low RP for men and women, and (iii) to determine the differences and substitution relationships between the configurations that lead to these outcomes.

For these purposes, this study proposes five propositions about the relationship between a set of conditions (national cultural and trust dimension) and two outcomes: female RP and male RP. Therefore, this essay uses fuzzy-set qualitative comparative analysis, a method that combines multiple cases and research depth properly, to explore pathways of retracted publications. Based on fsQCA, this essay examines how a country's cultural and trust dimensions (causal conditions) interact to cause high (or low) RP (outcomes) for both men and women. The method of fsQCA expands our understanding of what factors influence RP between men and women, and reveals the essence of RP by configuration. A total of 30 countries were included in the sample.

This essay is divided into five parts. A secondary part follows this introduction, which covers literature review and theoretical framework, and then the data, variables, and methods for analysis. An empirical study is then carried out by collecting data from 30 countries in order to obtain measurement results. The final sections will deal with the discussion, conclusion, and implication, providing appropriate policy recommendations aimed at cultivating publication integrity by exploring and comparing cultural dimensions and trust behind male and female RP nationally and globally.

## Literature background and theoretical framework

### National culture and RP

The determinants that cause RP can be analyzed at multiple levels, such as at the individual or micro level, or at the macro level. The culture of a country is a major factor affecting RP. Recognizing the influence of cultural factors makes it clear how RP shows specific national characteristics and comparative global analysis. To understand the influence of different cultures on retracted papers, studying cultural pathways showing in different countries offer a unique opportunity to explore different national cultures in a global context, where both local culture and intercultural factors play a role.

Cross-cultural issues allow researchers to include variables that are not normally considered, such as the importance of religion, language, race, or history, to provide new insights (Song, 2009). Scholars' and scientists' mindsets and behaviors are profoundly influenced by culture, affecting their preferences when conducting scientific research activities (Kassim et al., 2019). Retraction is influenced henceforward by cultural tradition, in which social and cultural background cannot be ignored (Mousavi & Abdollahi, 2020). The comparative understanding of the contexts of countries is essential for exploring both routes to cultivating publication integrity and preventing publication misconduct.

This essay explores the relationship between RP and cultural dimensions and trust in different countries. Under this context, we introduce Hofstede's five-dimensional cross-cultural comparative model as the theoretical basis, which has been extensively applied (Deephouse et al., 2016). According to Hofstede, culture is a collective pattern formed in people's minds that can distinguish members of different groups or categories. It depicts the impact of deep-rooted culture on the values of social members and provides a scoring system for dimensional comparison (Minkov & Hofstede, 2011). Five dimensions of this model are discussed below.

As a first step, Hofstede defines five basic cultural values: power distance, individualism, masculinity, uncertainty avoidance, and long-term orientation. The power distance expresses the degree to which the less powerful members of a society accept and expect unequal distribution of power. Individualism measures how people in society value individuals or groups. Masculinity is characterized by achievement, heroism, self-confidence, and material success (Cui et al., 2020). An individual's degree of discomfort with uncertainty and ambiguity is expressed by uncertainty avoidance. A long-term orientation considers whether people's behavior is based on their current needs or on their long-term development.

Regarding the relationship between national cultural dimension and RP, some conclusions have been reached. National cultures that emphasize individualism tend to view RP as individual preferences and choices (Tang, 2022). Cultures with a low degree of uncertainty avoidance are more tolerant of abnormal behavior, emphasizing less preference for establishing unified rules and absolute beliefs (Matusitz & Musambira, 2013). Furthermore, honest scholars value their reputation and long-term development, and do not touch the bottom line of RP (Nevins et al., 2007). In contrast, scientists concerned with their current state of acquisition tend to emphasize the potential benefits of RP. In this research, the following propositions are made:

#### Proposition 1

None of the national culture dimensions is a necessary condition to predict high RP for males.

#### Proposition 2

None of the national culture dimensions is a necessary condition to predict high RP for females.

#### Proposition 3

There are combinations of national culture dimensions that are sufficient to predict high RP for both males and females.

### Trust

Generally, trust is viewed as a relationship between persons or groups of persons based on the belief that the other party is trustworthy and well-meaning. The degree of trust between individuals is correlated with RP in some studies. Research suggests that trust has a positive effect on scientific activities (Frankel & Ebrary, 2005). Science is based on trust, which is a necessary condition for self-regulation of science to emerge and a catalyst for ensuring scholar-practitioners’ reputation (Madlock-Brown & Eichmann, 2015). Furthermore, the retraction of articles is an important part of retaining the trust of both the public and researchers in science (Resnik et al., 2015).

Firstly, within scientific research, there are many opportunities for collaboration and interpersonal interaction, and trust could facilitate more effective scientific research. Secondly, scholars rarely report the RP behavior of other scholars due to the relationship of trust between them, which further complicates the handling of such incidents. A social setting characterized by trust can serve as a framework for carrying out retractions, while at the same time encouraging publication integrity (Soehartono et al., 2022). Therefore, we use trusting attitude (whether we are willing to trust others) to measure the effect of social environment on RP. To conclude, these arguments show that:

#### Proposition 4

Some combinations of causal conditions that are sufficient to predict high RP for males or females include a high level of trust while others include a low level of trust (absence).

### Gender difference in RP

Most researchers use gender to describe research behavior, and find that there are fewer women conducting scientific research than men. Although the number of women engaged in scientific research has risen in recent years, there is still a significant gap between men and women in the number of women participating in science (Chimba & Kitzinger, 2010). The reality is, female scientists have to take care of their families and children, so they spend less time on scientific research. Indirectly, this leads to fewer female scientists working in scientific research. Besides that, researchers admit that men and women have different values and thinking styles. They adhere to different codes of conduct, have different research experiences and networks, and conduct research in different fields. The world is understood by female scientists in a more perceptive and delicate way. While this is true, we still have a limited understanding of the factors that influence the incidence of RP differently in men and women.

Differences between male and female RP are well-documented as well. In the past few decades, gender has been an important topic for RP's research. Compared to male scientists, fewer female scientists are engaged in RP. Men are more likely than women to retract publications and fraud and plagiarism accounted for more men-authored retractions (Decullier & Maisonneuve, 2021). Male scientists are more likely to engage in RP, which may reflect aggressiveness, competitiveness, the pursuit of status and the spirit of adventure men possess (Fang et al., 2013). Silva et al (2021) analyze the authorship characteristics of retracted COVID-19 articles and find there are more first authors and last authors male than female. According to some scholars, female scientists feel more resources related to responsible research behavior, possibly because they care more about procedural justice and resource allocation than men do (Haven et al., 2019).Researchers in the scientific community should be more aware of the pitfalls about retracted papers and deconstruct the bias on gender (Abdel-Razig et al., 2021).

RP becomes more complex when gender and scientific research are intertwined and must incorporate more cultural and social factors to unravel the complexity of such issues. In the same context, when the observation dimension concentrates on an individual, the micro-level factors become more relevant. Since the purpose of this study is to explore observational phenomena at the national dimension, it focuses on the national cultural and trust dimensions in order to reveal the complex structures that contribute to high (or low) RP for both males and females. These arguments show that:

#### Proposition 5

There are combinations of causal conditions (national culture dimensions and level of trust) that are sufficient to predict high (or low) RP specifically for either males or females.

## Method

### Data

Three sources of data were used to gather the data for this study. Firstly, data on RP for men and women comes from Web of Science, which now includes a column for retracted publications. The search strategy is to search for articles in the core collection under the title "retracted". The search date is September 15, 2021. Using the 7156 pieces of data retrieved from Web of Science, we use Python's gener api to determine the author's gender. In the case of authors with accuracy rates less than 50 and unable to be identified by API, two authors manually searched researchgate, google scholar, and various URLs. In total, 6371 retractions were found in men and women across different countries.

Fang and Casadevall (2011) define a “retraction index” for each journal as the number of retractions in the time interval from 2001 to 2010, multiplied by 1000, and divided by the number of published articles with abstracts. Besides that, Marcus and Oransky (2017) review some articles about retractions that these studies only focused on the number of retracted articles and did not take into account the total number of papers published in these countries, making the conclusions lack of robustness. Learning from the literature, we use the female and male RP data and divided by the number of published articles in Web of Science.

Secondly, trust is an important factor affecting retractions. Therefore, we use the World Value Survey questionnaire to measure trust variable. The questionnaire refers to the question that "Generally speaking, would you say that most people can be trusted or that you cannot be too careful in dealing with people?". This data disclosed from 1981 to 2022, and waved every four years. Considering the retracted publications was collected by September 15, 2021, we collected and averaged the data from 1981 to 2022 as trust variable.

Thirdly, Hofstede's cultural dimensions theory is a framework proposed by Geert Hofstede to measure cultural differences in different countries. He believes that culture ranks among the strongest influences on human behavior. Culture is a psychological program shared by people in an environment, which can distinguish a group of people from others. Through research, he summarized the differences between different cultures into six basic dimensions of cultural values. The last dimension, indulgence, was added in the book in 2010. We collect data on initial five dimensions: power distance, individualism, masculinity, uncertainty avoidance, and long-term orientation, do not include the last dimension in the model. The national cultural dimension data, which is a constant not involving time, is revealed in the Hofstede Insights Website.

Both homogeneity and heterogeneity are considered in the selection of case samples (Mazanec et al., 2015). From the perspective of geography, we selected 30 countries across the globe, including Asia and Oceania (China, India, Iran, South Korea, Australia, Saudi Arabia, Singapore, Thailand, Vietnam, Jordan), North America (the United States, Canada), Europe (France, the United Kingdom, Netherlands, Sweden, Norway, Russia, Romania, Hungary, Bulgaria), Latin America and Caribbean (Brazil, Mexico, Argentina, Chile, Trinidad Tobago), Africa (Egypt, South Africa, Nigeria, Morocco). The cultural backgrounds of selected case countries are heterogeneous, which meets the diversified criteria of case selection. In addition, 30 countries reflect more or less retraction under social culture context, which meets the homogeneity requirement for case selection.

### Condition and outcome variable

Outcome variables include male RP (RPm) and female RP (RPf), which means retraction of an article whose first author is a male or female. Condition variables include: (i) national culture dimensions: all of the dimensions—power distance (PDI: low versus high), individualism (IND: collectivism versus individualism), masculinity (MAS: femininity versus masculinity), uncertainty avoidance (UAI: low versus high), and long-term orientation (LTO: short-term versus long-term)—are all indices that range from 0 to 120. Score close to 0 represents low power distance, collectivism, femininity, low uncertainty avoidance, and short-term orientation, while score close to 120 means high power distance, individualism, masculinity, high uncertainty avoidance, and long-term orientation. (ii) trust, this variable affects scholars' behavior, which is considered to have an effect on RP. The data are shown in Appendix Table 1.

### FsQCA method

This essay aims at identifying the cultural factors that contribute to RP across different countries. To conduct an empirical analysis, fuzzy-set qualitative comparative analysis (fsQCA), a method that focuses on identifying causal conditions through configuration rather than net effects, is used. In the field of social science, causal relationships behind social phenomena are typically emphasized, which is exactly the core advantage of fsQCA in realizing causal inference. The fsQCA method can be applied to research to determine different causal combinations, thereby providing specific theoretical implications for the generation of results. Several scholars have favored and widely used this method as part of a "family of configuration comparison methods" (Lieberson & Ragin, 2001), which has been favored and widely used by many scholars. Hence, fsQCA has great potential in exploring the complexity of causality based on the breadth and depth of cases.

Utilizing fsQCA for this research has these benefits over traditional statistical analysis. Firstly, rich cases and in-depth analysis. With the combination of quantitative and qualitative methods, fsQCA not only allows horizontal comparison of several cases, but also enables deep context excavation within each case, thereby realizing both horizontal and vertical integration. Moreover, there are multiple ways to achieve the same outcomes simultaneously. There are two types of conditional relations toward outcome: sufficiency relation and necessity relation. Configuration and the conditional combination behind specific outcomes can be found through the iterative and creative process. Additionally, asymmetry of causality is used to explain why there is no one-to-one correspondence between the causal ways of high and low RP. Therefore, fsQCA yields more realistic perspectives on the mechanism behind RP, and accordingly, a better understanding of what to do about it by making appropriate policy recommendations.

FsQCA is mainly uses Boolean algebra and Boolean logic ("and", "not" and "or") to perform overall comparison. In practice, software fsQCA 3.0 is used to operate fsQCA. This method involves following process: original data are calibrated and compared under the same condition combination, and necessary conditions are analyzed to determine whether a single condition is a necessary condition to form outcome variable, then a specific truth table is constructed to show the causal condition combinations of different cases, and the next step is to build and analyze configurations. Therefore, the models used are as follow:

RP = f(PDI, IDV, MAS, UAI, LTO,TRU)

 ~ RP = f(PDI, IDV, MAS, UAI, LTO,TRU).^1^

#### Variable calibration

Because the fsQCA method is based on the set theory, it is necessary to use the calibration procedure to convert each research variable into a set, and the membership of the set after calibration will be between 0 and 1. The condition and outcome variables are considered as sets, and each variable has corresponding membership scores in these sets. The data are converted into fuzzy-set membership scores using the direct calibration method. As there is no clear theoretical and external knowledge to guide, and since the case countries are well represented, the calibration is based on the data of the case itself (Judge et al., 2020). Specifically, three thresholds (anchor points) of original data are set at 75% quantile (full subordination), 50% quantile (intersection), and 25% quantile (non-subordination) (Fiss, 2011). The calibration of the outcomes and conditions are shown in Appendix Table 2.

#### Analysis of necessary conditions

If a condition is a prerequisite for the occurrence of the outcome, it is a necessary condition. Consistency, as a measure of necessary conditions, reflects the extent to which cases with the same condition configuration contribute to the same outcome. Referring to the research of Schneider and Wagemann (2012), the necessary condition consistency threshold is set at 0.9. Appendix Table 3 shows the necessary conditions of high RP and low RP.

The consistency level of a single conditional variable is not greater than 0.9 for high or low RP, which indicates that variables do not constitute necessary conditions, or in other words, a single condition does not constitute a necessary element of retraction. From the perspective of cultural cultivation and change, the reasons for high and low RP are systematic and complex. The combined effects of power distance, individualism, masculinity, long-term orientation, and trust need to be explored further.

#### Build the truth table

The next step is to build a truth table that composed of multiple factors that lead to the outcome. Following the existing research, the configuration analysis consistency threshold is set to 0.85, the PRI consistency threshold is set to 0.8, and the frequency threshold is set to 1 (Alain et al., 2019). The full name of PRI is "Proportional Reduction in Inconsistency", which represents the degree of subset relationship between each condition and the outcome. In the truth table, each row represents a combination of conditions. Each individual condition can appear in the form of presence, 1, or absence, 0. In other words, the concept of "0" and "1" is to define whether a single dimension (column) in each case (each row) belongs to the set (1 means belonging, 0 means not belonging). Based on existing research (Fiss, 2013), this essay reports an intermediate solution, complemented by a parsimonious solution of fsQCA software output. Appendix Tables 4 and 5 show the truth tables for males and females without logical remainders respectively. Logical remainders are those rows without enough cases, so Appendix Tables 4 and 5 do not show those rows without enough cases.

## Results

### Results for high RP

The next step is to build and analyze the configurations. Appendix Table 6 shows the results of the fuzzy-set analysis for causing high RP for both females and males. Each column represents a different solution, grouped by male and female. Causality of different conditions is not consistent: in some configurations they exist (high value), whereas in others they are absent (low value), or they are dispensable. This result illustrates the diversity of conditions that could lead to high RP. Multiple combinations are associated with each configuration, and the multiple states presented by each condition are part of this outcome.

The results show that three configurations (High-Male 1, High-Male 2, and High-Male 3) are sufficient for causing high RP for men and three configurations (High-Female 1a, High-Female 1b, and High-Female 2) are sufficient for causing the same for women. The consistency of all these configurations is higher than the threshold of 0.8, and each configuration has five to six conditions (a total of six). Furthermore, all configurations of high RP for men and women include both core and edge conditions.

On one hand, High-Male 1 absent core conditions, showing high individualism, high uncertainty avoidance, and high long-term orientation, meanwhile presenting masculinity and absenting trust as edge conditions. And masculinity is an optional attribute. The consistency is 0.89 and coverage rate is 0.16. Trinidad Tobago is a representative country. High-Male 2 indicates the special configuration of women with high RP: uncertainty avoidance, long-term orientation, and trust present as core conditions, and masculinity and individualism absent as core and edge conditions respectively. The consistency is 0.92, the coverage is 0.15, and South Korea is a typical example of this type of case. High-Male 3 present high power distance, low individualism, low long-term orientation, low trust, and absent high uncertainty avoidance. The consistency is 0.92, the coverage is 0.06, and India is a representative country.

On the other hand, High-Female 1a and High-Female 1b share the same core conditions present or absent, which have high masculinity, low uncertainty avoidance, and high interpersonal trust. Besides that, High-Female 1a have low power distance, low individualism, and High-Female 2a have low individualism and low long-term orientation as edge conditions. This is sufficient to cause high RP for High-Female 1a and High-Female 1b, with consistency and coverage of 0.95, 0.28, and 0.95, 0.17, respectively. The United States, the United Kingdom, Australia and Canada are cased covered by High-Female 1a, and the United Kingdom, India, Canada are cases covered by High-Female 1b. In addition, High-Female 2 present high masculinity, low distance, low uncertainty avoidance, low long-term orientation, and absent high individualism. The consistency is 0.90, the coverage is 0.06, and Saudi Arabi is the country covered.

Analyzing the solutions for men and women from the perspective of consistency and coverage. According to intermediate solution, female RP has a consistency of 0.95, meaning 95% of 3 configurations cases present high female RP, and coverage of 0.36, which means that 3 configurations can explain 36% of the high female RP cases. And male RP has a consistency of 0.92, 92% of 3 configurations cases present high male RP, and coverage of 0.34, which means that 3 configurations can explain 34% of the high male RP cases. These cases provide equally effective solutions, showing women and men high RP configurations.

### Results for low RP

To fully understand the driving mechanism of high RP, we correlate the low RP with the causal asymmetry of the QCA method (i.e. the conditions leading to the appearance and absence of outcomes are not symmetrical). Appendix Table 7 shows configurations causing low RP.

The results show that two configurations (Low-Male 1 and Low-Male 2) are sufficient for causing low RP for men and three configurations (Low-Female 1a, Low-Female 1b, and Low-Female 2) are sufficient for causing the same for women. The consistency of all these configurations is higher than the threshold of 0.8, and each configuration has five to six conditions (a total of six). In addition, all configurations of low RP for men and women include both core and edge conditions.

On one hand, there are 2 low RP configurations for male. Low-Male 1 present high individualism, high long-term orientation, low trust, and absent high power distance, high masculinity, low uncertainty avoidance. The consistency is high (0.97), and the coverage rate is 0.19. In this configuration, Sweden and Netherland are prominent. Low-Male 2 show 3 core conditions, high masculinity and high long-term orientation exist and low trust absents. And at the same time, 3 edge conditions are analyzed, low power distance, low individualism and low uncertainty avoidance exist and low individualism absents. The consistency is 0.87, the coverage is 0.07, and Brazil is the country covered.

On the other hand, 3 low RP configurations are available for female. There are 2 configurations share the same core conditions that cause low RP for women: Low-Female 1a and Low-Female 1b, a culture of high masculinity, high uncertainty avoidance, high trust. Besides that, for Low-Female 1a, low individualism, low long-term orientation is absent, and for Low-Female 1b, low power distance and low long-term orientation is absent. Among two configurations, consistency is 0.91 and 0.90, while coverage is 0.16 and 0.13 respectively. And country cases covered are Trinidad Tobago, Nigeria, and Trinidad Tobago, South Africa respectively. Low female 2 is characterized by presenting high individualism, high masculinity, high uncertainty avoidance, high long-term orientation as core conditions and low trust as edge condition, meanwhile absenting low power distance as edge condition. The consistency is 0.88, and the coverage is 0.06. Hungary is a representative sample.

Analyzing the solutions for men and women from the perspective of consistency and coverage. According to intermediate solution, male RP has a consistency of 0.95, meaning 95% of 2 configurations cases present low male RP, and coverage of 0.24, which means that 2 configurations can explain 24% of the low male RP cases. And female RP has a consistency of 0.90, 90% of 3 configurations cases present low female RP, and coverage of 0.24, which means that 3 configurations can explain 24% of the low female RP. These cases provide equally effective solutions, showing women and men low RP configurations.

## Discussion and conclusion

The phenomenon of retractions in scientific research makes it imperative to study this field. This study aims to perform a causal analysis that measures the possible combined effects of cultural and trust dimensions on a country's male and female RP, exploring the causal complexity of both male and female RP across different countries. Hofstede's five-dimensional cross-cultural comparative model guides this essay as it examines combined influence mechanisms of national cultural (power distance, individualism, masculinity, uncertainty avoidance, long-term orientation) and trust dimensions on RP with 30 countries as research samples.

Both specific national and trust dimensions are not necessary to cause the outcomes, that is, one single condition cannot predict the high RP status for men or women, which supports propositions 1 and 2. Power distance, individualism, masculinity, uncertainty avoidance, long-term orientation, and trust dimensions cannot be necessary conditions of a country's high or low RP, suggesting that a single condition has weak explanatory power for RP. Therefore, RP requires a combination of several conditions to produce the outcome.

The phenomenon of retraction is complex. Combinations of different dimensions of national culture and trust can lead to high RP for both men and women, which supports proposition 3. There are some dimensions (power distance, individualism, masculinity, uncertainty avoidance, interpersonal trust) that show opposite states (existence or absence, high value or low value) in configurations that lead to high RP, regardless of gender. Power distance, for example, integrates 1 configuration to bring high RP to men, and 2 configurations to bring high RP to women. A loose atmosphere and low power distance, as advocated by the principle of collective openness, can enhance publication integrity and reduce retracted articles of research misconduct (Anderson, 2007). The solutions of female high and low RP show that masculinity always has the same causal effect. Almost all the configurations show high masculinity. Regarding individualism, in high RP, it is half-existent and half-absent; in low RP, except it is optional in 1 configuration, it shows the same result. This means that the cultural values of individualism and collectivism have a relatively consistent impact on RP. Trust exists as a core condition in high or low RP, but it exists in high RP and is absent in low RP. This further response previous literature with clear conclusion, and also verifies proposition 4.

Based on the results, 3 configurations including causal conditions were sufficient to predict high male or female RP, explaining 34% of the outcome for male and 36% of the outcome for female. For male, power distance integrates 1 configuration, all other conditions integrate 3 configurations. For female, power distance and long-term orientation integrate 2 configurations, and other 4 conditions integrate 4 configurations. In regard to the non-set of high RP (low RP), there are 2 configurations sufficient to predict low male RP and 3 configurations to predict low female RP, explaining 24% of the outcome for male and female. For male, 6 conditions integrate all configurations. For female, power distance and individualism integrate 2 configurations, and other 4 conditions integrate 3 configurations. These conclusions raise challenges over other studies (Embleton & Helfer, 2007), which generally discuss the factors that affect both males and females in the same way. However, based on the causal exploration and causal asymmetry of fsQCA, we conclude in this article that some conditions that lead to male and female RP are completely different from one another. Proposition 5 is fully supported.

The contributions of this essay are as follow. Firstly, this essay examines RP from the perspectives of both men and women. There is an inherent inconsistency between men and women when it comes to conducting research and retracting papers (Decullier & Maisonneuve, 2021; Silva et al., 2021). Various data analyses should be conducted on differences between male and female RP (Crespo, 2017; Lewellyn & Muller-Kahle, 2016). Therefore, this study examines the cross-country RP differences between men and women from a structural perspective. According to our research, male authors have retracted more manuscripts than female authors in countries with the exception of Argentina, which largely confirms existing research findings (Smyth & Davis, 2004). Secondly, the study fills in gaps in the interaction or configuration of cultural dimensions between some researchers (Shamsi, 2020). Through Hofstede's theory and the cross-cultural comparative analysis framework presented in this article, we are able to improve the effectiveness and practice of this theory in the field of RP. Thirdly, this study enlarges the scope of RP to explicitly include both national and global perspectives simultaneously. Researchers may get a better understanding of the retraction of the country through this study, which can then be used in policy-making to encourage publication integrity.

### Implication for theory

In some ways, this essay contributes to the exploration and improvement of RP. Firstly, as part of Hofstede's intercultural comparative analysis model, we explore five key elements as "cultural variables", as well as trust as an important "environmental variable". The integrated analysis framework is helpful to understand the macro situation that affects RP. This essay verifies five cultural and trust dimensions on RP, which provides a theoretical basis for an empirical analysis of the synergistic effect of conditions on RP.

Secondly, from the perspective of configuration, this essay empirically investigates the synergistic effects of power distance, individualism, masculinity, uncertainty avoidance, long-term orientation, and trust on RP, thus expanding the applications of different national examples to causal complexity of RP. The theory of cross-cultural comparative analysis is widely applied to the comparison of different countries, but it rarely involves RP. On this level, this study provides insight into complex mechanisms of multi-cultural interaction factors affecting RP, while simultaneously extending the application of cross-cultural comparative theory in analyzing the influence of several factors on RP.

Thirdly, this essay introduces fsQCA method, which offering a new approach for identifying the cultural influences that contribute to low and high RP in men and women, into the field of RP and deepens understanding of the complex causal mechanisms that affect RP. The fsQCA method, based on a holistic perspective, is an excellent tool for understanding the complex phenomenon of RP, which has rarely been used currently in the field of RP. By introducing fsQCA into the national and global levels, this essay clarifies the multiple combined conditions that lead to high RP in different countries as well as the underlying mechanism that drives low RP from the standpoint of "causal asymmetry", adding to the application field of fsQCA.

### Implication for practice

These findings are of great importance for public policy makers, as they offer policy implications as follows to promote publication integrity and curb publication misconduct in a country. Firstly, when formulating public policies (such as journal or university policies) that circumvent RP, the cultural background of the country should be considered. The cultural features of each country are complex and multi-leveled. Therefore, forming public policies cannot consider only a single cultural level, but must take into account the national culture as a whole. In some cultures, male and female RP are circumvented in the different way. To increase policy effectiveness, public policies should differentiate between female and male.

Secondly, RP in a country is affected by many factors. A government cannot take all factors into account at the same time under limited resources, therefore some necessary goals must be targeted. Nevertheless, since the development and cultivation of culture is a long-standing process, the government can tailor some dimensions of culture to a more suitable direction according to original specific combinations. In this essay, it is noted that multiple factors can result a country's retraction through the pursuit of "the same goal in different ways". Therefore, in the case of limited resources, a country should consider combining its advantages to choose the appropriate cultural combinations that will fit its endowment, optimizing key factors and compensating for insufficient development factors to ensure publication integrity.

### Limitation and further research

There are some limitations to the study. Firstly, the data on the cultural dimension of this essay was collected from the Hosftede Center using his typology of cross-cultural comparative analysis. Using the classification of culture types developed by other scholars (Trompenaars & Hampden-Turner, 2011) and the Globe Project (House et al., 2002) may help test the validity of the conclusions of this research.

Secondly, due to the limited availability of data, this essay is focused on a comparative analysis of six conditions toward RP, without considering dynamic changes in variables over time. As a future research direction, we can consider the dynamic evolution of the cultural mechanism of retraction.

Thirdly, men and women RP are divided in an autonomous manner. Creating new sources directly related to these two variables, such as Scopus, Pubmed, and Retraction Watch, may be a supplementary analysis. If other sources of data on male and female RP become available, relevant research can be conducted to compare with the findings.

## Appendix

See Tables 1, 2, 3, 4, 5, 6 and 7.

**Table 1:** Description of condition and outcome variables, statistical sources and descriptive statistics

	Index description	Data source	Descriptive statistics			
			Mean	SD	Max	Min
Outcomes						
RPm	Reracted publication by males/publication output of country	*Web of Science* Website	0.66	0.16	1	0.29
RPf	Reracted publication by females/publication output of country		0.02	0.05	0.23	0.00
Conditions						
PDI	Low status people's acceptance of unequal distribution of power in society or organization	*Hofstede Insights* Website	61.50	18.50	95	31
IND	Society as a whole pay more attention to individual interests or collective interests		46.03	24.93	91	16
MAS	The trait that represents men or women more obvious in society		47.63	17.79	88	5
UAI	Uncertainty avoided through formal channels when affected by uncertain events		61.60	22.13	95	8
LTO	Members pay more attention to long-term benefits or short-term favors		42.57	24.17	100	7
TRU	Trusting attitude (whether willing to trust others)	Trust Attitude report released by *World Value Survey*	31.04	16.06	70	4

30 countries include Asia and Oceania (China, India, Iran, South Korea, Australia, Saudi Arabia, Singapore, Thailand, Vietnam, Jordan), North America (the United States, Canada), Europe (France, the United Kingdom, Netherlands, Sweden, Norway, Russia, Romania, Hungary, Bulgaria), Latin America and Caribbean (Brazil, Mexico, Argentina, Chile, Trinidad Tobago), Africa (Egypt, South Africa, Nigeria, Morocco)

**Table 2:** Calibration of outcomes and conditions

	Calibration Criteria		
	Full subordination (75%)	Intersection (50%)	Non subordination (25%)
Outcomes			
RPm	0.76	0.68	0.61
RPf	0.0090	0.0030	0.0008
Conditions			
PDI	73.00	66.00	46.25
IND	70.50	38.50	25.00
MAS	60.00	48.50	40.00
UAI	82.00	61.50	48.25
LTO	57.75	36.00	21.75
TRU	40.00	26.41	19.25

**Table 3:** Summary of necessary conditions

	RPm				RPf			
	Presence		Absence		Presence		Absence	
	Consistency	Coverage	Consistency	Coverage	Consistency	Coverage	Consistency	Coverage
PDI	0.63	0.63	0.48	0.45	0.45	0.40	0.60	0.63
~ PDI	0.46	0.49	0.61	0.60	0.59	0.56	0.43	0.48
INDI	0.44	0.48	0.56	0.57	0.64	0.63	0.37	0.42
~ INDI	0.61	0.60	0.49	0.45	0.40	0.35	0.67	0.69
MAS	0.61	0.68	0.38	0.39	0.49	0.48	0.48	0.56
~ MAS	0.46	0.44	0.69	0.62	0.56	0.48	0.56	0.56
UAI	0.54	0.55	0.55	0.52	0.45	0.41	0.60	0.63
~ UAI	0.52	0.55	0.53	0.52	0.59	0.56	0.44	0.49
LTO	0.55	0.54	0.66	0.59	0.64	0.55	0.48	0.48
~ LTO	0.58	0.65	0.49	0.51	0.41	0.40	0.56	0.64
TRU	0.53	0.55	0.56	0.54	0.74	0.68	0.34	0.36
~ TRU	0.55	0.57	0.53	0.51	0.30	0.28	0.70	0.76

~ Means the not-set of condition variable

**Table 4:** Truth table for male without logical remainders

PDI	IND	MAS	UAI	LTO	TRU	RP	Raw consistency	PRI consistency
1	0	0	1	1	1	1	0.92	0.89
1	1	1	0	1	1	1	0.92	0.86
0	1	1	1	1	1	1	0.90	0.73
0	0	1	0	0	0	1	0.88	0.84
1	0	1	1	1	1	0	0.87	0.41
1	0	1	0	0	0	1	0.86	0.82
0	0	0	1	1	1	1	0.85	0.78
0	1	0	0	0	0	0	0.84	0.77
1	0	1	1	0	0	0	0.84	0.80
1	1	1	1	0	0	0	0.83	0.76
0	1	1	0	0	0	0	0.82	0.48
0	0	0	1	0	1	0	0.76	0.59
0	1	1	0	1	1	0	0.74	0.61
1	0	0	0	1	1	0	0.63	0.46
1	0	0	1	0	0	0	0.59	0.50
1	1	0	1	1	0	0	0.59	0.36
0	0	0	1	0	0	0	0.58	0.42
0	1	1	0	0	1	0	0.54	0.20
1	0	1	1	1	0	0	0.53	0.22
0	1	0	0	0	1	0	0.50	0.35
0	1	1	1	0	0	0	0.49	0.23
1	0	0	1	1	0	0	0.36	0.25
0	1	0	0	1	1	0	0.18	0.01

**Table 5:** Truth table for female without logical remainders

PDI	IND	MAS	UAI	LTO	TRU	RP	Raw consistency	PRI consistency
0	1	1	0	1	1	1	0.94	0.93
0	1	1	0	0	1	1	0.93	0.92
1	0	1	1	1	1	1	0.90	0.84
1	1	1	0	1	1	1	0.90	0.84
0	1	0	0	1	1	0	0.84	0.82
1	0	1	1	1	0	0	0.81	0.72
0	1	0	0	0	0	0	0.79	0.70
0	0	0	1	1	1	0	0.73	0.66
1	1	0	1	1	0	0	0.66	0.60
0	1	0	0	0	1	0	0.64	0.53
1	0	0	1	1	1	0	0.62	0.55
0	0	0	1	0	1	0	0.50	0.38
1	0	0	1	0	0	0	0.49	0.42
0	0	0	1	0	0	0	0.45	0.35
0	1	1	0	0	0	0	0.42	0.20
0	1	1	1	0	0	0	0.39	0.24
0	1	1	1	1	1	0	0.38	0.16
1	1	1	1	0	0	0	0.38	0.21
1	0	1	1	0	0	0	0.34	0.22
1	0	0	0	1	1	0	0.34	0.23
1	0	1	0	0	0	0	0.33	0.16
0	0	1	0	0	0	0	0.32	0.13
1	0	0	1	1	0	0	0.29	0.21

**Table 6:** Configuration for causing high retracted publication

	Males			Females		
	High-Male 1	High-Male 2	High-Male 3	High-Female 1a	High-Female 1b	High-Female 2
PDI			●	△		○
IND	▲	△	○	○	○	▲
MAS	○	▲	●	●	●	●
UAI	▲	●	▲	▲	▲	○
LTO	▲	●	○		○	○
TRU	△	●	○	●	●	●
Consistency	0.89	0.92	0.92	0.95	0.95	0.90
Raw coverage	0.16	0.15	0.06	0.28	0.17	0.06
Unique coverage	0.14	0.14	0.03	0.16	0.04	0.04
Overall solution consistency	0.92			0.95		
Overall solution coverage	0.34			0.36		
Case	Trinidad Tobago	South Korea	India	the United States, the United Kingdom, Australia, Canada	the United Kingdom, India, Canada	Saudi Arabi

● Indicates the core condition exists

○ Represents the edge condition exists

▲ Indicates the core condition does not exist

△ Represents the edge condition absent, and the blank indicates the condition is dispensable. Core conditions are defined as conditions with strong causal relationship with outcome variables, while edge conditions are those with weak causal relationship with outcome variables. Configuration High-Female 1a and High-Female 1b indicate they share the same core condition present or absent

**Table 7:** Configuration for causing low retracted publication

	Males		Females		
	Low-Male 1	Low-Male 2	Low-Female 1a	Low-Female 1b	Low-Female 2
PDI	▲	○		△	△
IND	●	△	△		●
MAS	▲	●	●	●	●
UAI	△	○	▲	▲	●
LTO	●	●	△	△	●
TRU	○	▲	▲	▲	○
Consistency	0.97	0.87	0.91	0.90	0.88
Raw coverage	0.19	0.07	0.16	0.13	0.06
Unique coverage	0.18	0.05	0.07	0.03	0.04
Overall solution consistency	0.95		0.90		
Overall solution coverage	0.24		0.24		
Case	Sweden, Netherland	Brazil	Trinidad Tobago, Nigeria	Trinidad Tobago, South Africa	Hungary

● Indicates the core condition exists

○ Represents the edge condition exists

▲ Indicates the core condition does not exist

△ Represents the edge condition absent, and the blank indicates the condition is dispensable. Core conditions are defined as conditions with strong causal relationship with outcome variables, while edge conditions are those with weak causal relationship with outcome variables. Configuration Low-Female 1a and Low-Female 1b indicate they share the same core condition present or absent

## Footnotes

1 ~ RP refers to the non-set of RP.
